# Novel Anti-Apoptotic MicroRNAs 582-5p and 363 Promote Human Glioblastoma Stem Cell Survival via Direct Inhibition of Caspase 3, Caspase 9, and Bim

**DOI:** 10.1371/journal.pone.0096239

**Published:** 2014-05-07

**Authors:** Desiree Hunt Floyd, Ying Zhang, Bijan K. Dey, Benjamin Kefas, Hannah Breit, Kaitlyn Marks, Anindya Dutta, Christel Herold-Mende, Michael Synowitz, Rainer Glass, Roger Abounader, Benjamin W. Purow

**Affiliations:** 1 Division of Neuro-Oncology, Departments of Neurology, Microbiology, and Biochemistry and Molecular Genetics, University of Virginia Health System, Charlottesville, Virginia, United States of America; 2 Division of Neurosurgical Research, Department of Neurosurgery, University of Heidelberg, Heidelberg, Germany; 3 Charité University Clinics, Clinic for Neurosurgery, Berlin, Germany; 4 Neurosurgical Research, University Clinics Munich, München, Germany; University of Michigan School of Medicine, United States of America

## Abstract

Glioblastoma is the most common and lethal primary brain tumor. Tumor initiation and recurrence are likely caused by a sub-population of glioblastoma stem cells, which may derive from mutated neural stem and precursor cells. Since CD133 is a stem cell marker for both normal brain and glioblastoma, and to better understand glioblastoma formation and recurrence, we looked for dys-regulated microRNAs in human CD133+ glioblastoma stem cells as opposed to CD133+ neural stem cells isolated from normal human brain. Using FACS sorting of low-passage cell samples followed by microRNA microarray analysis, we found 43 microRNAs that were dys-regulated in common in three separate CD133+ human glioblastomas compared to CD133+ normal neural stem cells. Among these were several microRNAs not previously associated with cancer. We then verified the microRNAs dys-regulated in glioblastoma using quantitative real time PCR and Taqman analysis of the original samples, as well as human GBM stem cell and established cell lines and many human specimens. We show that two candidate oncogenic microRNAs, miR-363 and miR-582-5p, can positively influence glioblastoma survival, as shown by forced expression of the microRNAs and their inhibitors followed by cell number assay, Caspase 3/7 assay, Annexin V apoptosis/fluorescence activated cell sorting, siRNA rescue of microRNA inhibitor treatment, as well as 3′UTR mutagenesis to show luciferase reporter rescue of the most successful targets. miR-582-5p and miR-363 are shown to directly target Caspase 3, Caspase 9, and Bim.

## Introduction

Glioblastoma multiforme (GBM) is a devastating brain tumor with an average survival time from diagnosis of 12–14 months [1]. Malignant primary brain tumor morbidity in the US is around 22,000 cases per year, and 80% are GBMs [2]–[4]. Surgical resection, followed by radiation and chemotherapy with temozolomide (Temodar), is the current standard of care [5]. However, GBMs invariably recur after a short period of remission. Recent studies reveal tissue heterogeneity in human brain tumors, and there is accumulating evidence that tumor initiation, recurrence, and the low rate of survival are likely dependent upon a small fraction of glioblastoma stem cells (GSCs) that are chemo- and radio-resistant [6]–[11].

GBMs are thought to arise from transformation of neural stem cells (NSCs) or de-differentiation of mature glioma cells with genetic lesions [12], [13]. GSCs share some characteristics with neural stem and precursor cells (NSCs): expression of NSC/NPC markers such as CD133, Oct4, Nanog, Sox2, Nes, c-Myc, Mdr1 and Abcg2; self-renewal; multi-potentiality; and migratory capability [12]–[21]. There is accumulating evidence that the glioma cell-of-origin is a normal stem or precursor cell from the sub-ventricular zone. CD133 is one out of a few established markers that can indicate both NSCs and GSCs. CD133 may not be a universal marker for GSCs, and the validity of CD133 as a cell-surface molecule indicating glioma cells with stem-like properties has to be established for each individual tumor. We have previously used a range of *in vivo* and *in vitro* assays to establish that CD133 is a valid GSC marker for the human primary GBM cultures (GBM-2 or B4, NCH644 and NCH441) used in this study [22], [23].

microRNAs (miRNAs) are small noncoding regulatory RNA molecules with profound impacts on many biological processes. MicroRNAs (miRNAs) are coded both inter- and intra-genically. They are excised from longer RNAs and processed in the nucleus as short hairpin pre-miRNA, and are further processed in the cytoplasm into a double-stranded, 22-nucleotide RNA. One strand of the miRNA is then preferentially incorporated into the RISC protein complex, and this ribonucleoprotein complex binds to messenger RNAs and prevents their expression–but this normal processing is dys-regulated in cancer [24]. Recently, the discovery of miRNAs as drivers of tumor growth and survival has led to the evaluation of the miRNA expression pattern in different types of glioblastomas as compared to normal brain tissue [25]–[28]. miRNAs that are involved in neural development in NSCs have been shown to play a role in GBM development, and 70% of known miRNAs are expressed in the brain [26].

Some miRNAs that have been analyzed in GBM and other cancers have come to be characterized as oncogenic, or able to drive tumor growth through mechanisms such as treatment resistance, escape from the immune system or dys-regulation of angiogenesis. miRNAs that have come to be understood as oncogenic or tumor-driving, with their expression usually increased in GBM, include but are not limited to miR-21, the miR cluster 17–92, miR-26a, and miRs-221 and -222 [29]–[33]. The well-studied oncogenic miRNAs in GBM tend to decrease expression of genes that are known to be tumor suppressors in GBM; for example, miR-21 targets well-known tumor suppressors such as PTEN [25], [28].

Tumor suppressor miRNAs are defined as those that decrease growth or invasion and are usually down-regulated in GBM. We and others have shown that miR-7 and miR-34a suppress well-known oncogenic targets, such as EGFR, c-Met, and Notch family members [34]–[38]. In some cases, despite the association of a miRNA with tumor-suppressive function, the key targets are unknown, as in the case of miR-124 and miR-137 [39]. Several of the miRNAs that drive or suppress GBMs have similarly oncogenic or tumor-suppressive properties in other cancers [25], [28], but it should be noted that even with the well-established oncogenic miR-21, there is controversy regarding the behavior of the miRNA toward its known or potential targets, and therefore the effect of the miRNA upon cell proliferation [30]–[32], [40], [41]. It is also important to note that miRNAs dys-regulated in one GBM are not necessarily dys-regulated in another, and therefore our data analysis is weighted toward discovery of miRNAs that are frequently dys-regulated in GBM stem cells as compared to normal brain stem cells.

A recent work published miRNA expression levels in GBM stem cells compared to normal NSCs by identifying entire cell lines as pluripotent based on the ability of some of the cells to differentiate into multiple cell types [42]. This approach identified previously known dys-regulated miRNAs, but did not identify novel miRNAs. We asked whether novel miRNAs dys-regulated specifically in GBM stem cells would be revealed by comparing several samples of low-passage CD133-positive GSCs to low-passage normal human CD133-positive NSCs. Using this approach, we successfully identified novel microRNAs that are dys-regulated in GBM. After studying the tumor-specific microRNAs using microarray analysis and validation in many GBM cell lines and human specimens, we studied forced expression of the antagonists of miR-363 and miR-582-5p, finding that in both GBM stem cells and other cancers, the miR inhibitors caused an up-regulation of apoptosis and vastly increased cell death, indicating that GBMs up-regulate certain oncogenic miRNAs in order to suppress apoptotic targets. We confirmed several apoptotic targets of miR-582-5p and miR-363 including Caspase 3, Caspase 9, and Bim using immunoblot and luciferase reporter assays including rescue of luciferase reporter signal with 3′UTR mutagenesis, and we also performed a phenotypic rescue using siRNA against the targets in the context of miR inhibition.

## Materials and Methods

### Ethics Statement

Primary human GBM cultures enriched for GSCs termed GBM-2 (originally B4), NCH644, and NCH441 were previously published [22], [23], [43], and were initially obtained from surgical resections from GBM patients ( written “informed consent was originally obtained according to the research proposals approved by the Institutional Review Board at the Medical Faculty Heidelberg”; The University of Virginia received the isolated RNA samples, and analysis was performed without identifying information), as previously described [22], [23], [43].

### Cells and Tissues

Primary human GBM culture enriched for GSCs termed 0308, 0822, and 1228 were previously published [44] and obtained from surgical resections from GBM patients (“Following informed consent, tumor samples classified as GBM based on World Health Organization (WHO) criteria were obtained from patients undergoing surgical treatment at the National Institutes of Health in accordance with the appropriate Institutional Review Boards”) [44]. Further information in the Supporting Information. Extensive information about the characterization of the human NSCs is also provided in the Supporting Information.

GBM stem cell lines (0308, 0822, and 1228) were obtained from Dr. Jeongwu Lee [44] and maintained as previously described [36], [37], [44]. For the purposes of experimentation, cells to be transfected with miRNA or miRNA inhibitors were either plated in dishes with the transfection mixture, or induced to adhere with poly-L-ornithine and laminin and then transfected the next day. There were not substantial differences in the response of the GBM stem cell lines to the miRNA inhibitor with either plating technique.

GBM lines U373 and A172 were obtained from the American Type Culture Collection repository and was maintained as previously described [34], [45]. Immortalized astrocytes were a gift from Russell Pieper.

Human specimen RNA from eleven GBMs and three normal human brain samples were obtained from surgical samples and cDNA was generated to evaluate miRNA levels in these specimens.

### Flow Cytometry of Neurospheres for Microarray Analysis

Fluorescence-activated cell sorting (FACS) analysis was carried out on a LSR II (BD Biosciences, Erembodegem, Belgium). Cell sorting was carried out using a FACS-Aria-II (BD Biosciences). Antibodies human CD133 and human isotype control were all from Miltenyi Biotech (BergischGladbach, Germany). Data were analyzed using CellQuestand FACSDiva (BD Biosciences) and FlowJo software (Treestar,Ashland, OR, USA).

### Microarray and Analysis

All microRNA arrays were performed twice and initial analysis was performed by Beckmann Technologies, using the human microRNA version 2 (GPL7731) platform. Full analysis was performed for four samples: B4 human glioma CD133–, B4 human glioma CD133+, BLV4 human NPC CD133–, and BLV4 human NPC CD133+. Secondary analysis was performed for CD133+ NCH644 and NCH441 samples. Raw data and metadata were deposited in the NCBI GEO database, Accession numbers are: GSM1199638–GSM1199643 respectively. The microarray heatmap shown was created using Java Treeview. Raw intensity values were transformed by Beckman Coulter into log base 2 fold change intensity ratios for each CD133+ GSC sample compared to the NPC CD133+ sample, and each partial data analysis was provided as a separate Excel spreadsheet. miRNAs that demonstrated an initial p-value of less than.05 were then further selected if two GBM CD133+ samples demonstrated at least 0.7 log fold change in intensity ratios in the same direction (increased or decreased), as in other similar analyses where the 95% confidence interval falls around this line [46]. The final p-values for the miRNAs selected for the heatmap were all less than.001 for two of three GSC samples. Microsoft Excel spreadsheets of the submitted GEO raw data, as well as analyzed data, showing selection criteria, log fold intensity changes compared to CD133+ NSCs, and p-values are shown in the supporting information in the **Files S3, S4,** and **S5**. Previously unstudied miRNAs that met these criteria were selected for experimental follow-up.

### Quantitative PCR

Select miRNAs were validated by quantitative PCR in a variety of tissue and cell types. Template RNA from the CD133+ NSCs and CD133+ GBM stem cell samples was prepared. Human specimen sample RNA was prepared using miRNeasy and miScript RT kits (Qiagen, Valencia, CA), according to the protocol directions. cDNA was diluted to 10 ng/microliter and 50 to 100 ng of cDNA was used per reaction. Primer assays (Qiagen, Valencia, CA) were used for each miRNA tested. 2 X Sybr Green reagent from ABI was used for qPCR. The control primer set was RN_U6B, recommended by Qiagen for miRNA analysis.

### microRNAs, siRNAs, and Transfection

Validated microRNAs were purchased as pre-miRNAs from Ambion (Grand Island, New York), and used with the recommended Ambion pre-miRNA control. Anti-miRs and a scrambled Anti-miR control for validated oncogenic miRNAs were also purchased from Ambion. miRNA transfections were carried out using Oligofectamine (Invitrogen, Grand Island, New York) reagent as previously described [36].

### Immunoblots

Protein expression of miRNA targets was evaluated by immunoblot as previously described [34], [45]. Primary antibodies were rabbit anti-Caspase 3 and mouse anti-Caspase 9 (Cell Signaling, Danvers, MA), mouse anti-alpha-tubulin (Sigma Aldrich, St. Louis, MO), anti-BCL2L11 (2933S, Cell Signaling, Danvers, MA), anti-beta-actin (Santa Cruz Biotechnology, Santa Cruz, CA). Protein content of the samples was normalized to alpha-tubulin (51 kDa) or beta-actin (43 kDa). For miR-363, illustrated bands were quantified using a histogram in Adobe Photoshop, and for miR-582-5p, all available blots were quantified in Image J using the Gel Analysis tool, and full-length scans are supplied as examples in **Figure S6 in File S2**. Western blots were repeated 2–7 times with multiple transfected wells per experiment.

### Cell Counting

Cell number was evaluated to determine the effect of forced miRNA or miRNA inhibitor transfection. Cells were counted three to five days after miRNA transfection. Ten microliters were analyzed using a hemocytometer.

### Caspase 3/7 Assay

Caspase activity was detected as a means to evaluate apoptosis in cells expressing miRNAs or miRNA inhibitors. Cells were assayed for Caspase 3/7 activity three to four days after miRNA transfection using Caspase 3/7 Glo substrate solution as recommended (Promega, Madison, WI). Luminescence was measured using a Glomax luminometer (Promega, Madison, WI). Luminescence readings were corrected by cell number and also compared to protein concentration via Bradford assay (Pierce, Rockford, IL). Values shown are RLU/cell number per mL, normalized to a percent maximal activity.

### Flow Cytometry of Apoptotic Cells

Total apoptotic cell percentage was evaluated using a dual marker flow cytometry kit. Cells were stained four to five days after miRNA transfection using the PE-Annexin-V Apoptosis staining kit I (BD Pharmingen, San Diego, CA), as recommended. Positive cells were sorted using a FACSCalibur and analyzed with FlowJo software.

### Caspase Inhibition

A broad caspase inhibitor was used to rescue the effect on cell numbers of miRNA inhibitor driven up-regulation of Caspase proteins. Cells were transfected with miRNA and then treated with 40 µM Z-VAD-fmk (Caspase Inhibitor I, CalBiochem, San Diego, CA) or vehicle for three days prior to cell count.

### 3′-UTR Luciferase Assays

Luciferase reporting of miRNA target 3′-UTRs was used to determine specificity of the miRNA-target mRNA interaction. Caspase 3-3′-UTR-RenillaLuc was designed and initially tested by us, and other 3′-UTR-luc plasmids for BCL2L11 (GeneCopoeia, Rockville, MD) and Caspase 9 (Origene, Rockville, MD) were used to define specific targets of the miRNAs. Cells were plated as described above and transfected with miRNA and then the appropriate plasmid. Plasmid transfection was carried out using Fugene HD (Roche, Indianapolis, IN) and was paired with a transfection control plasmid encoding beta-galactosidase (Origene, Rockville, MD). Forty-eight hours post-plasmid transfection, luciferase activity was assayed using Promega Renilla Luciferase Assay System or Promega Firefly Luciferase Assay system (Madison, WI). Luciferase activity was corrected using beta-galactosidase activity and normalization with control-miRNA and empty vector controls.

### 3′-UTR Mutagenesis

Primers encoding the entire seed match region of the 3′-UTR for BCL211, CASP3, and CASP9, were designed with 5–8 base pair substitutions introduced in the strongest putative binding region for the miRNA, and an Agilent Site Directed Mutagenesis kit (Santa Clara, CA) was used to introduce these mutations into the wild-type 3′-UTR of the targets. Two miR-363 binding sites, one for BCL211 and one for CASP3, were mutagenized, and the binding region for CASP3 for miR-582 was also mutagenized. The primer sets were as follows: For the 5′ CASP 3 site for miR-582: Forward: 5′CCCCCCACTTAAGACTGTGTATTCTAGTTT. TGTCAA*CGGCGGC*AAATGATGATGTGG 3′. Reverse: 5′ CCACATCATCATTT*GCCGCCG*TTGACAAAACTAGAATACAC. AGTCTTAAGTGGGGGG 3′, with wild-type sequence: 5′ CCCCCCACTT AAGACTGTGT ATTCTAGTTT TGTCAAACTG TAGAAATGATGATGTGG 3′.

For the miR-363 binding site in BCL2L11 (Bim) 3′UTR, Forward: 5′ CTTATCAACTGAGCCAAATGTCTGT*CGCGCCGG*GTGTTTCCTTTACCTTGTAAAATTTTG 3′, Reverse: 5′ CAAAATTTTACAAGGTAAAGGAAACAC*CCGGCGCG*ACAGA CATTTGGCTCAGTTGATAAG 3′; with wild-type sequence: 5′ CTTATCAA CTGAGCCAAATGTCTGTGTGCAATTGTGTTTCCTTTACCTTGTAAAATTTTG 3′. For the miR-363 binding site in CASP 3′UTR, Forward: 5′ AAATTAGGAATAAATAAAAAT GGATACTG*CGCGCCG*CATTATGAGAGGCAATGTTGTTAA 3′, Reverse: 5′ TTAACAA CATTGCCTCTCAT AATG*CGGCGCG*CAGTATCCATTTTTATTTATTCCTAATTT 3′ with wild type sequence: 5′ AAATTAGGAATAAATAAAAATGGATACTGGTGCAGTCA TTATGAGAGGCAATGATTGTTAA 3′.

Further methods are available in **Methods S1**.

## Results

### miRNAs are Differentially Expressed in CD133+ GSC Samples Versus Normal CD133+ NSCs

**Figure 1:**
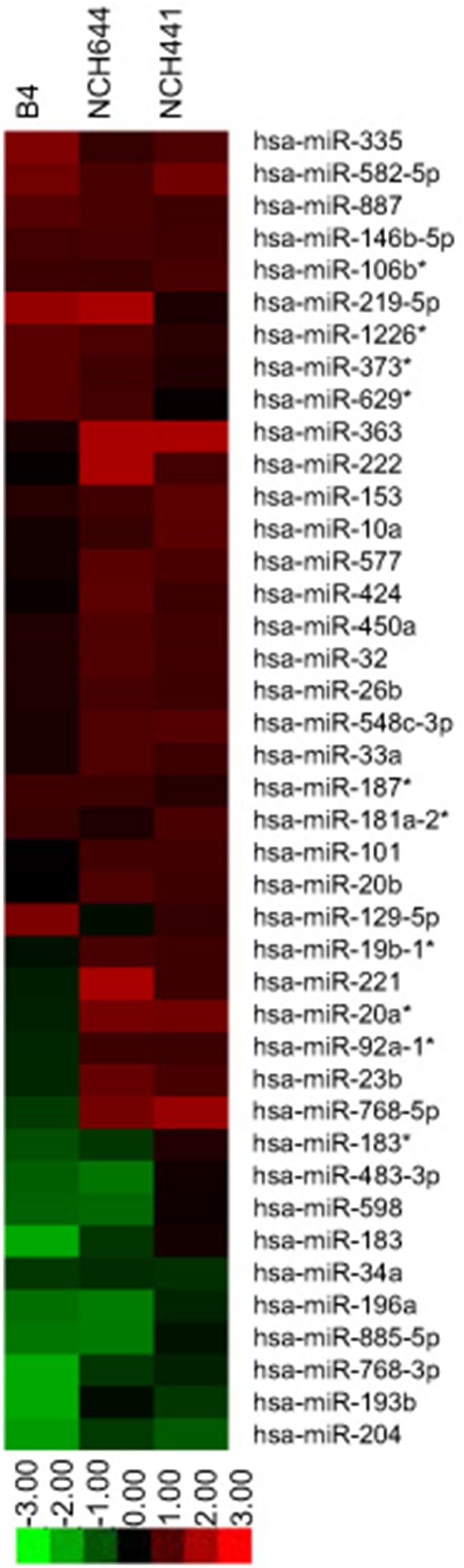
Microarray heatmap of significant miRNA changes. Relative normalized (log) fold intensity changes for miRNAs in CD133+ GBM sample populations B4, NCH644, and NCH441 are shown in comparison to CD133+ NSCs. Increased miRNAs are shown in red; decreased miRNAs are shown in green.

Using microarray analysis, we measured miRNAs in FACS-sorted CD133+ (B4, NCH644, and NCH441) and – human GSC samples (B4) and CD133+ and – normal human neural stem cells (BLV4). We then further evaluated those miRNAs that were strongly increased or decreased only in CD133+ GSC relative to CD133+ NSCs (**Figure 1**). Five miRNAs were strongly increased in all three CD133+ GSC samples via this selection method, and a total of thirty-one miRNAs were increased in at least two out of three CD133+ GSC samples. The novel up-regulated miRNAs were miR-582-5p, miR-363, miR-577, and miR-887, which have not been previously associated with any cancer. Curiously, miR-219-5p, miR-33a, miR-101, and miR-129-5p have been identified in at least one other cancer as tumor suppressors [47]–[50], and miR-146b-5p has been described as preventing invasiveness in glioma [51], but in CD133+ GSCs these miRs are increased. Several miRNAs from known oncogenic miRNA clusters, such as the miR-17∼92 cluster and miR-221/222, were strongly up-regulated [52]–[55].

Eleven miRNAs were decreased in CD133+ GSCs, with two miRNAs strongly decreased in all three samples. miR-768-5p, miR-768-3p, and miR-598 are novel decreased miRNAs (mir-768 is no longer in the TargetScan database, due to its relationship with its host snoRNA and confusion about sequence conservation). miR-483-3p and miR-196a have been previously identified as being oncogenic in other cancer settings, but they were decreased in at least 2 of our GSC samples [56], [57].

All other dys-regulated miRNAs in this study have been identified as dys-regulated in at least one other tumor type, but few except for the very well-known miR clusters have been identified previously in GBM. miR-34a has been extensively studied by our group and others as a tumor suppressor miRNA in GBM and other brain tumors [34], [58]–[60], and it was found to be decreased in all three CD133+ GSC samples. miRNAs that have little prior association with oncogenesis or tumor suppression, or were otherwise previously unstudied, represented about 30% of the total identified miRNAs in the microarray.

### miR-363 and miR-582-5p are Strongly Upregulated in GSC Samples, Multiple GBM Human Tumor Specimens, and GBM Neurosphere Stem Cell Lines

**Figure 2:**
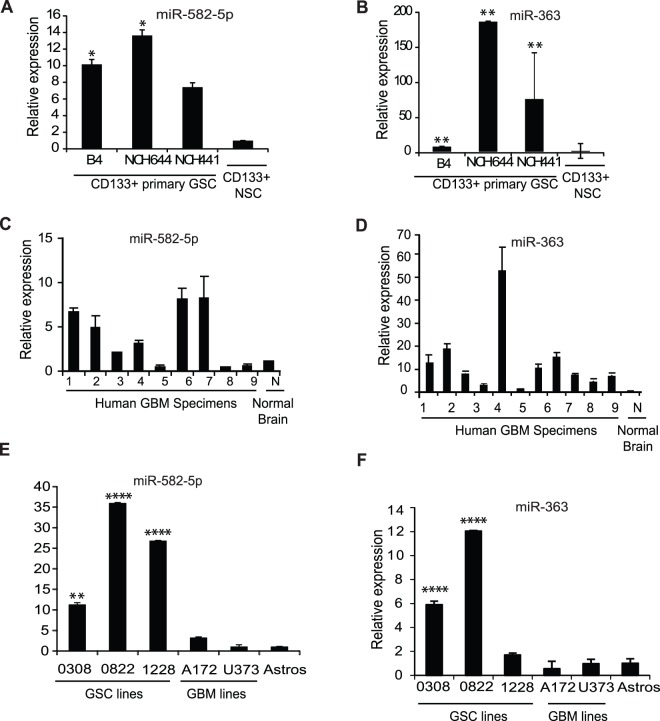
Real-time PCR verification of miR-582-5p and miR-363 up-regulation in GSCs. Relative expression of miR-582-5p and miR-363 are depicted for qPCR confirmation of the experimental CD133+ microarray GSC and NPC samples (A and B). Relative expression of miR-582-5p and miR-363 in nine separate human tumor specimens compared to an average of three normal human brain tissue controls with three technical replicates per sample (C and D). Baseline relative expression of miR-582-5p and miR-363 in established (A172 and U373) and stem-like GBM cell lines (0308, 0822, and 1228), as well as normal astrocytes (E and F). An average of two technical replicates and three separate RNA/cDNA samples are shown. Column-to-column p-values are given for A, B, E, and F, taken from one-way analysis of variances followed by Bonferroni’s post-test to compare all columns. Significant differences are indicated thus: *, p<.05, **, p<.01, ****, p<.0001.

In order to confirm the initial results from the microarray, we performed Taqman and traditional quantitative PCR analysis of miRNA expression in the CD133+ NSCs and the three CD133+ GSC samples. miRNAs that were differentially expressed versus the NSCs in all three GSC samples were given priority. Most miRNAs recapitulated the data in the microarray (**Figure S1 in File S1**). miR-582-5p and -363 were chosen for further analysis via quantitative PCR (qPCR) due to their novelty as potentially oncogenic miRNAs, and the strength of their relative fold changes (**Figure 2A and 2B**). miR-582-5p has been shown to be over-expressed in pituitary adenomas, and is predicted to target Smad-3, but is otherwise unstudied [61]. miR-363 has not been specifically studied in cancer, though it lies within a known oncomiR cluster [62], [63]. To further delineate the level of expression of the miRNAs in GBM tumor versus normal tissue, we next measured miR-582-5p and miR-363 via qPCR in many GBM and three normal human tissue specimens. We found that miR-582-5p was increased in six GBMs, up to ten fold relative to the normal samples (**Figure 2C**). In three specimens miR-582-5p was decreased or undetectable (and therefore not shown), indicating that despite the increased expression of miR-582-5p in the CD133+ GSC sample population, not all GBM tumors up-regulate miR-582-5p. miR-363 was increased, sometimes to extremely high levels, in nine human specimens as compared to three averaged normal brain samples (**Figure 2D**).

Levels of miR-582-5p and miR-363 were next assessed in three previously published GBM stem cell lines, which have been maintained in neurobasal medium with supplements and without serum (0308, 0822, and 1228) (**Figure 2E and F**) [44]. These lines and others like it have been shown to be more similar to original GBMs than cell lines maintained in serum [6], [44], [64], [65]. We also tested the levels of miR-582-5p and miR-363 in GBM lines that have been maintained in serum, A172 and U373 (**Figure 2E–F**) and others, (**Figure S2A–B in File S1**) as well as immortalized astrocytes, by traditional qPCR and/or Taqman. The established oncomiR miR-221 was also measured in all of the cell lines to reconfirm the pattern of higher expression of oncomiRs in the GBM stem cell lines (**Figure S2C in File S1**). miR-582-5p and miR-363 were strongly upregulated in all available GSC samples, most human GBM specimens, and the GBM stem cell lines.

### miR-582-5p and miR-363 Regulate GSC Growth and Apoptosis

**Figure 3:**
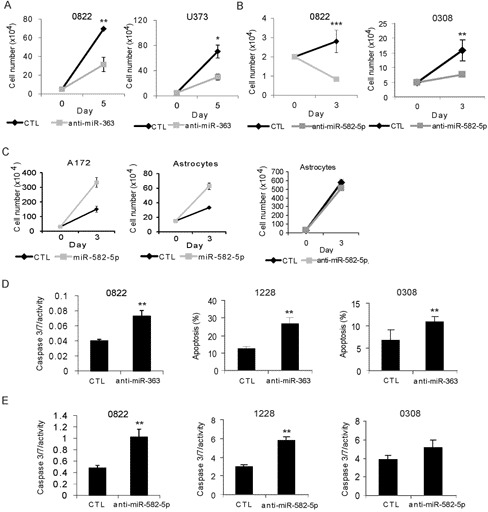
Forced expression of anti-miR-363 and anti-miR-582-5p triggers apoptosis. 0822 and U373 GBM stem cell numbers for anti-miR-363 (grey squares), compared to scrambled anti-miRNA negative control (black diamonds) (A). 0822 and 0308 cell numbers for anti-miR-582-5p (grey squares), compared to scrambled anti-miRNA negative control (black diamonds) (C). Immortalized astrocyte and established GBM cell line A172 cell numbers following transfection of miR-582-5p (grey squares) compared to control miRNA (black diamonds), as well as for anti-miR-582-5p (grey squares) compared to anti-miRNA control (black diamonds) for immortalized astrocytes (C). Caspase 3/7 activity and/or Annexin V positivity (shown as % apoptosis) for anti-miR-363 in GBM stem cell lines 0308, 0822 and 1228, compared to scrambled anti-miRNA negative control (D). Caspase 3/7 activity for GBM stem cell lines for anti-miR-582-5p, compared to scrambled anti-miRNA negative control (E). p-values for Student’s t-test analyses are given as *, p<.05, **, p<.01, ***, p<.001. Experiments shown are representative of at least three separate experiments per cell type.

Evaluation of the biological effects of miR-582-5p and miR-363 through forced expression of their specific inhibitors (stabilized short antisense inhibitor molecules) in GBM neurosphere stem cell lines and serum-maintained GBM lines confirmed their oncogenic potential. anti-miR-363 caused growth-inhibition of the GBM stem cell line (0822) and the established cell line U373 (**Figure 3A**). Two GBM stem cell lines (0822, 0308) treated with anti-miR-582-5p demonstrated growth inhibition of 50% compared to control-inhibitor-treated cells (**Figure 3B**), whereas a serum-maintained GBM line, A172, and immortalized human astrocytes treated with miR-582-5p itself exhibited faster growth (**Figure 3C**). Growth of an immortalized human astrocyte line treated with anti-582-5p was unchanged in comparison to scrambled anti-miR control (**Figure 3C**). These data are consistent with the hypothesis that miR-582-5p and miR-363 are oncogenic. We next established that growth inhibition of GBM stem cell lines via anti-miR treatment was a direct result of increased apoptotic cell death. Anti-miR-363 treated cells exhibited higher Caspase 3/7 Glo activity or Annexin V/7-AAD expression in all GBM stem cell lines (**Figure 3D**). There was a 2- to 3-fold increase in Caspase 3/7 Glo activity in anti-miR-582-5p treated GBM stem cells (**Figure 3E**). We also observed a decrease in apoptotic activity in miR-treated cell lines (**Figure S4 in File S2**). These results support an anti-apoptotic mechanism of action of miR-363 and miR-582-5p.

### miR-582-5p and miR-363 Regulate Pro-apoptotic Gene Expression by Directly Targeting Caspase 3, Caspase 9, and Bim 3′-UTRs

**Figure 4:**
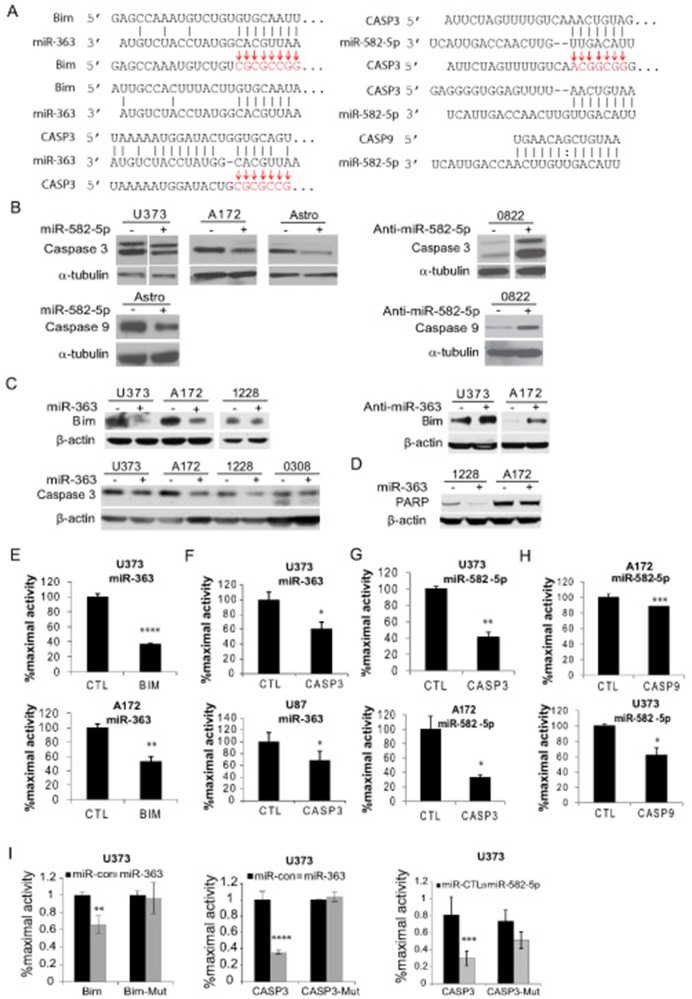
miR-582-5p and miR-363 target Caspases and Bim. Seed match regions for miR-363 and miR-582-5p in the 3′UTRs of Bim (BCL211), Caspase 3 (CASP3), and Caspase 9 (CASP9), with arrows over mutagenized bases in 3′UTR mutant constructs (A). Immunoblots of Caspase 3 and Caspase 9 for miR-582-5p- or anti-miR-582-5p, three to four days post-transfection of miR-582-5p (+) or control miRNA (–), or anti-miR-582-5p (+) or control scrambled anti-miR (–) Full blots are provided in the supplemental data for single lanes shown, depicting several samples per blot which represent separate transfection wells (B). Immunoblots of Bim and Caspase for miR-363- or anti-miR-363, three to four days post-transfection of miR-582-5p (+) or control miRNA (–), or anti-miR-582-5p (+) or control scrambled anti-miR (–) (C). PARP cleavage immunoblot with forced expression of miR-363 (D). 3′UTR luciferase reporter activitymiR-363 for Caspase 3Bim, or control plasmid (E–F), and for miR-582-5p for Caspase 3 and Caspase 9 or control plasmid (G–H), after correction with control miRNA and control plasmid to yield % maximal activity. 3′-UTR luciferase reporter activity for wild-type and mutagenized BIM and CASP3 target 3′-UTR putative binding sites for miR-582-5p or miR-363 compared to control miR (I). p-values for two-column experiments are derived from Student’s t-tests and for multi-column experiments are derived from two-way ANOVA, with *, p<.05, **, p<.01, ***, p<.001, ****, p<.0001. All blots are representative of several (three or more) separate experiments/transfections, with two to three wells per group per experiment.

In order to understand the biological relevance of miR-582-5p and miR-363, we evaluated potential targets. miR-363 was predicted to target Bim (BCL2L11) and Caspase 3 by TargetScan seed match analysis, and miR-582-5p was predicted to target Caspase 3 and Caspase 9 (**Figure 4A**). We also show in **Figure 4A** the residues mutated in 3′UTR mutagenesis experiments, delineated by arrows. miR-582-5p-treated GBM cell lines U373 and A172 as well as astrocytes expressed less Caspase 3 and Caspase 9 as shown by Western blot (**Figure 4B**, densitometry averages of 2–7 lanes per graph shown in **Figure S3B in File S1**, full blot exposure examples for single lanes that were cut from originals are shown in **S6A–C in File S2**, results were variable but were repeated several times for each cell line). In keeping with this, the 0822 GBM stem cell line treated with anti-miR-582-5p expressed higher Caspase 3 and Caspase 9 as compared to anti-miR scrambled control (**Figure 4B, S** **3B in File S1,** and **S6 in File S2**). GBM cell lines treated with miR-363 expressed less Bim and Caspase 3 protein, whereas GBM lines treated with anti-miR-363 expressed more Bim protein (**Figure 4C**, bands shown were quantified by densitometry in **Figure** **S3A in File S1**). The increases in target protein expression with anti-miR delivery indicate that these miRs are potent physiological regulators of Caspase 3 and Bim, respectively. A qPCR analysis of putative miR-582-5p targets confirms Caspase 3 and Caspase 9 mRNA expression sensitivity to this miR, and indicates that Caspase 10 may also be a target (**Figure S5A in File S2**). miRNA target mRNAs and protein levels are not always consistently regulated, however, and we were unable to show consistent response of Caspase 10 at the protein level [66]. We also show less cleaved PARP with miR-363 forced expression in two cell lines, an indicator of decreased apoptotic activity (**Figure 4D**). The interaction of miR-582-5p and miR-363 with the predicted target 3′-UTRs was shown to be specific via luciferase reporter assays in multiple GBM lines, with normalization using control miRNA and empty plasmid, for BIM and CASP3 for miR-363 **(** **Figure 4E–F** **, Figure** **S5B in File S2**), and for CASP3 and CASP9 for miR-582-5p **(** **Figure 4G–H**). Mutagenesis of putative 3′UTR binding sites for the miRNAs resulted in a partial rescue of luciferase reporter activity in the presence of the miRNAs compared to control scrambled miRNA and wild-type 3′UTR plasmids (**Figure 4I**). These data suggest that miRNAs expressed by tumor stem cells effectively down-regulate key apoptotic pathway proteins.

### Caspases and Bim are Critical Targets of miR-582-5p and miR-363, Respectively

**Figure 5:**
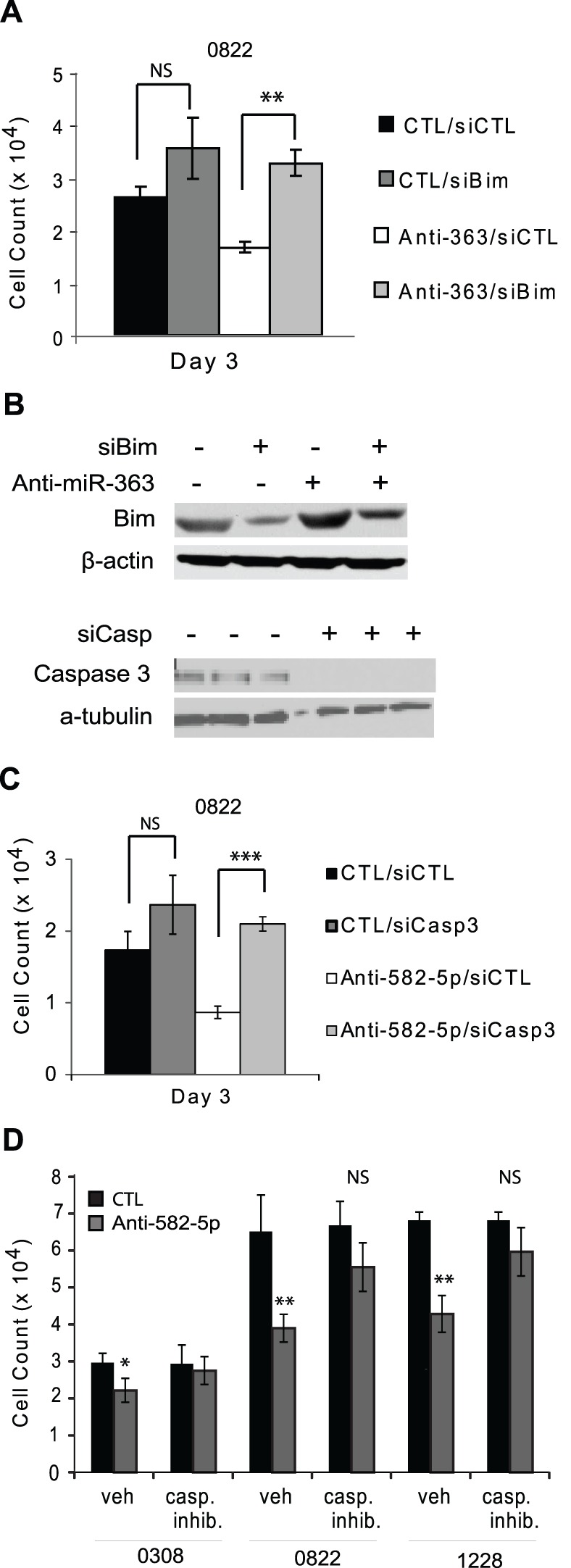
Caspase 3 and Bim are critical targets of miR-582-5p and miR-363. 0822 cell numbers Bim siRNA and anti-miR-363 rescue experiment with si- and anti-miRNA scrambled controls (A). Bim immunoblot for similar conditions (B). Caspase 3 immunoblot with siRNA treatment (B) as well as 0822 cell numbers for Caspase 3 siRNA and anti-miR-582-5p rescue experiment with si- and anti-miRNA scrambled controls (C). GBM stem cell numbers following transient transfection with anti-miR-582-5p (grey columns) compared to anti-miRNA scrambled controls, with ZVAD caspase inhibitor treatment rescue (D). One-way ANOVA with Bonferroni’s post-test to compare all columns was used to generate p-values. p-values are represented as *, p<.05, **, p<.01, ***, p<.001 Experiments are representative of three or more separate experiments per panel with up to six separate wells per group.

To test the importance of Caspase and Bim targeting in the effects of miR-582-5p and miR-363 on cell numbers, we performed Caspase and Bim inhibition combined with anti-miR expression. The cell number decrease that was expected for populations treated with anti-miR-363 was prevented by RNA silencing of Bim (**Figure 5A**). As expected, the increase in Bim expression observed with forced expression of anti-miR-363 was prevented with co-expression of Bim siRNA (**Figure 5B**). Similar results were obtained for co-expression of anti-miR-582-5p and siRNA targeting Caspase 3; an immunoblot of cells treated with Caspase 3 siRNA demonstrates the effectiveness of the siRNA (**Figure 5B and 5C**). In addition, an experiment to test the response of anti-miR-582-5p-expressing GSCs to a pan-Caspase inhibitor, ZVAD, resulted in a partial rescue of cell numbers in the presence of the Caspase inhibitor (**Figure 5D**). These results demonstrate that suppression of Caspases and Bim by these miRs is critical to their effects on cancer cells.

## Discussion

Because of the unclear mechanisms of GSC formation and regulation, there is a critical need to study the role and therapeutic potential of miRNAs in GSCs. A few studies of miRNAs have investigated GSC regulation [34], [36], [38], [67], [68], but no global analysis has previously been performed using GSCs compared with NSCs isolated from human tissues using CD133 as a selective marker. The present study reveals significant differential expression of miRNAs in three GSC samples in comparison to normal NSCs (adult NSCs are difficult to obtain), including both previously studied (such as miR-34a and miR-221/222) and novel miRNAs.

Beginning with this approach, we further studied two novel anti-apoptotic miRs, miR-363 and miR-582-5p. We show that miR-363 and miR-582-5p are highly expressed in many human GSC populations and GBM tumor specimens. We found that miR-363 and miR-582-5p directly target the apoptotic mRNAs Caspase 3, Caspase 9, and Bim. miR-363 and miR-582-5p behave as oncogenes, causing increased growth in immortalized astrocytes and well-established GBM cell lines, while the antagonism of miR-363 and miR-582-5p results in apoptotic cell death and decreased cell numbers in GBM stem cell lines. We rescued the effects of miR-363 and miR-582-5p antagonism with knockdown of Bim and Caspase 3, respectively, showing them to be the key targets of these oncomiRs in cancer.

Although the cancer stem cell hypothesis is becoming better established, roles for miRNAs in cancer stem cells are little-studied. While these results suggest a number of miRNAs that may play important roles in cancer stem cells, we focused on two miRNAs that protect these cells from apoptosis. The self-renewal and proliferation of stem cell populations is partially regulated by induction of apoptosis. The number of stem cells is therefore a balance between those lost to differentiation/apoptosis and those gained through proliferation. Dys-regulation of apoptosis in stem cells is believed to underlie some cancer pathologies, where apoptotic resistance results in uncontrolled growth [69].

The mechanisms by which miR-363 and miR-582-5p down-regulate apoptosis seem clear. Bcl-2 and Caspase family members play major roles in cell apoptosis. The well-known Caspase cleavage cascade begins with initiator Caspase activation by intrinsic or extrinsic pathways. Caspase 9 is an intrinsic initiator caspase, and its activation occurs at the mitochondrial membrane with the release of cytochrome C; Caspase 3 is an effector caspase that mediates the cleavage of many cellular proteins in tandem with other effector caspases [70]. The role of miR-582-5p in down-regulating two caspase cascade components is thus clearly anti-apoptotic.

**Figure 6:**
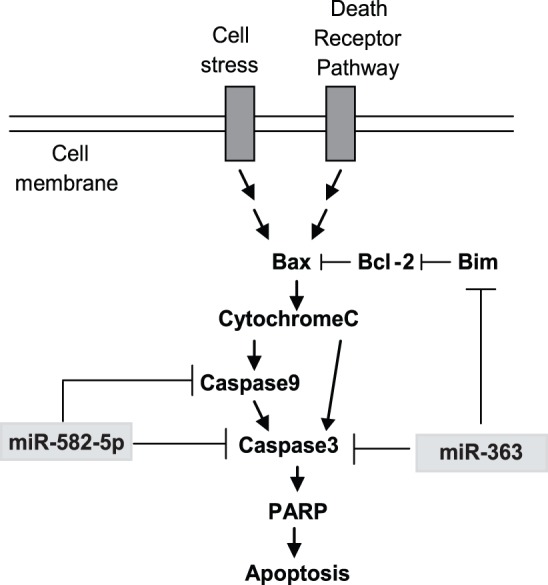
The apoptotic cascade. A graphical representation of the miRNAs 582-5p and 363 targeting and decreasing expression of multiple apoptotic pathway components.

The Bcl-2 family consists of a number of structurally related proteins that regulate the intrinsic apoptosis pathway by controlling mitochondrial membrane permeability and the release of cytochrome c. The BH3-only pro-apoptotic protein BCL2L11 (Bim) regulates apoptosis by binding to anti-apoptotic members of the Bcl-2 family, including Bcl-2, Bcl-xl and Mcl-1, or by activating pro-apoptotic members Bax and Bak (**Figure 6**) [71], [72]. We show here that miR-363 regulation of Bim prevents GBM stem cell apoptosis, enhances Bcl-2 expression, and decreases PARP cleavage. Furthermore, miR-363 also directly targets Caspase 3 to enhance the effects on apoptosis inhibition in GSCs. miR-582-5p targets the initiator Caspase 9 as well as the effector Caspase 3, thus dismantling the intrinsic apoptotic pathway.

This report also demonstrates that up-regulation of apoptotic pathway target proteins is sufficient to increase apoptotic activity in the case of both these novel miRNAs. While specific up-regulation of full-length Caspase-3 has been shown to make T-cells susceptible to apoptosis [73], it has not been studied in the context of miRNA regulation or in cancer. Bim is often down-regulated by mitogens such as ERK, and FOXO3a expression is known to induce Bim, but apoptotic induction through increased Bim expression after anti-miR treatment has not been previously demonstrated.

The strong up-regulation of both miR-363 and miR-582-5p across many GSC samples and their co-targeting of intrinsic apoptotic pathway components suggest that GSCs have a susceptibility to mitochondrial membrane destabilization and apoptosis. GSCs may attempt to evade apoptosis by increasing expression of these miRNAs. Several current anti-tumor therapies induce apoptosis in cancer cells using antibody delivery of toxic molecules, antibody binding to death receptors such as TRAIL followed by apoptotic induction, and inhibition of histone deacetylases (HDACs) leading to apoptotic induction [74]–[76]. Tumor initiation and progression is likely partly accomplished in GBM through apoptotic dys-regulation; it has been reported that apoptotic dysregulation in hematopoietic stem cells (HSCs) can lead to differentiation defects and increased cancer risk [77–78]. Our results suggest a novel method for inhibiting tumor growth, via antagonism of the anti-apoptotic oncomiRs miR-582-5p and -363. Progress is being made with local delivery of siRNAs and miRNAs or miRNA inhibitors for brain tumor therapy, and anti-miR-582-5p and anti-miR-363 may represent potent payloads. This work establishes miR-582-5p and miR-363 as important physiologic drivers of GSC resistance to apoptosis, providing new points of therapeutic leverage against these treatment-resistant cells.

## Supporting Information

File S1 **Contains the files: Figure S1: Taqman qPCR of several possible miRNAs increased or decreased in CD133+ GBM primary cultures compared to CD133+ NSCs.** miRNA-specific reverse transcription, followed by Taqman primer and probe amplification of miRNA targets is shown as Taqman Fold Change for miRNAs with little or no prior association to GBM. **Figure S2: Further quantification of miR-582-5p, miR-363, and known oncogenic miR-221 in GBM cell lines.** miR-582-5p fold change in GBM established cell lines T98G, U87, and U251, relative to level of the miR in normal astrocytes (A). miR-363 fold change in GBM established cell lines T98G, U87, and U251, relative to level of the miR in normal astrocytes (B). Level of miR-221 in many GBM stem and established cell lines, normalized to the cell line with the lowest level, A172 (C). **Figure S3: Densitometry of immunoblots.** Bim, Caspase 3, and Parp protein in several cell lines was quantified using an Adobe Photoshop pixel intensity histogram and normalized to corresponding alpha-tubulin protein bands from the same lanes in immunoblots of control scrambled miR, miR-363, control scrambled anti-miR, and anti-miR-363 treated cell lysates (A). Caspase 3 and Caspase 9 protein in 2–7 lysate samples was quantified using Image J gel analysis tool by calculating the area under the curve for each band, normalized to alpha tubulin from the same lane, and averaged for all quantified blots (B).
(EPS)

File S2 **Contains the files: Figure S4: Reduced apoptosis with miR-582-5p forced expression in established GBM cell lines.** Caspase 3/7 luciferase activity (A) and Annexin V staining (shown as % apoptosis (B) in immortalized astrocytes and U373, an established GBM tumor cell line, respectively, in the presence of miR-582-5p or control miRNA. Student’s t-test was performed to generate p-values, with ****, p<.0001. **Figure S5: Further miR-363 and miR-582-5p data showing targets related to apoptosis.** mRNA levels of predicted targets were measured after forced expression of control scrambled anti-miRNA (black bars) or anti-miR-582-5p (grey bars) to determine whether the miR had a possible direct effect on mRNA levels of targets (A and B). Additional data showing normalized empty vector vs. Caspase 3 3′UTR reporter activity response to miR-363 (C) in U87, an established GBM cell line. *, p<.05. **Figure S6: Western blots that were cut to show representative lanes are shown in full.** In the case of U373 and 0822 cells, single lanes cut from the larger blots of Caspase 3 and alpha-tubulin are shown in Figure 4, so examples of full blot exposures are shown here (A and B). The 0822-Caspase 9 blot shown in (C) is re-loaded from samples that needed correction of the loading control.
(EPS)

File S3 **Supplemental Microarray File 1:** A Microsoft Excel document showing tables of p-values and log-fold intensity ratios for CD133+ GSCs as compared to CD133+ NSCs for selected miRNAs for the heatmap in Figure 1.
(XLSX)

File S4 **Supplemental Microarray File 2:** Raw intensity values for all miRNAs and samples tested in a Microsoft Excel document, also all raw data files are available online at GEO under accession numbers GSM1199638–GSM1199643.
(XLS)

File S5 **Supplemental Microarray File 3:** A Microsoft Excel document showing selection criteria for miRNAs in the microarrays.
(XLSX)

Methods S1 A Microsoft Word document detailing some protocols and reagents.
(DOC)
