# Supplementary material for: Novel Anti-Apoptotic MicroRNAs 582-5p and 363 Promote Human Glioblastoma Stem Cell Survival via Direct Inhibition of Caspase 3, Caspase 9, and Bim
Source: PLoS One. 2014 May 7;9(5):e96239. doi: 10.1371/journal.pone.0096239 (PMC4013090; doi:10.1371/journal.pone.0096239)
Supplement: Methods S1 — A Microsoft Word document detailing some protocols and reagents. (DOC) [file pone.0096239.s006.doc]

*Supplemental Experimental Procedures*

*Characterization of human NPCs*--For the experiments of the present study neurospheres were analyzed after maximally 15 passages in vitro. The cultures could be maintained for more than 50 passages in vitro without apparent loss in plating efficiency. After 20 passages in vitro we purified genomic DNA from our cultures and studied the integrity of the genome after in vitro expansion by performing array-comparative genomic hybridisation. We did not observe any chromosome aberrations in cultured NPCs. Single cell suspensions of neurospheres were plated onto Laminin (Invitrogen) and Ornithine (Sigma) coated glass coverslips and maintained as a single layer of adherent NPCs in NB-medium plus additives (see above) for three days.

Adherent NPCs were analyzed by immunohistochemistry with antibodies for: goat anti-Doublecortin (Santa Cruz Biotechnology, Heidelberg, Germany); mouse anti nestin (Santa Cruz Biotechnology); guinea pig anti-GFAP (AdvancedImmunoChemical, Long Beach, CA); rabbit anti-Ki67 (Novocastra Laboratories Ltd., Newcastle, UK); rabbit anti-NG2 (Chemicon, Temecula, CA); rabbit anti-S100β (Swant, Bellinzona, Switzerland); rabbit anti-Musashi (Chemicon); mouse anti-PSA-NCAM (Chemicon); monoclonal mouse anti-O4 (Chemicon); and rabbit anti-TUC-4 (Chemicon). FITC- , RhodX- or Cy5-conjugated secondary antibodies (all from Jackson ImmunoResearch Laboratories, West Grove, PA) were used. Immunofluorescent double labeling was performed and recorded using a spectral confocal microscope (Leica TCS SP2). Appropriate gain and black level settings were determined on control tissues stained with secondary antibodies alone.

*Cell lines used only in the Supplemental Results*—U87 and U251 were purchased from the American Type Culture Collection repository and were maintained as previously described. XO-4 and XO-8 are glioma stem cells kindly provided by Dr. Deric Park (Department of Neurosurgery, University of Virginia, Charlottesville, VA), and were maintained in DMEM F12 with EGF, FGF, and B27.

*MicroRNAs, siRNAs, and transfection*— Validated microRNAs were purchased as pre-miRNAs from Ambion (Grand Island, New York), and used with the recommended Ambion pre-miRNA control. Anti-miRs for validated oncogenic miRNAs were also purchased from Ambion. miRNA transfections were carried out using Oligofectamine (Invitrogen, Grand Island, New York) reagent as previously described (Kefas et al., 2009)with a final miRNA concentration of 20 nM. The transfection mix was added to the cells and incubated for five hours, and then cells were fed with NBM or appropriate medium. For experiments lasting longer than three days, cells were re-fed with new medium on the third day post-transfection.

*Immunoblots—* Protein expression of miRNA targets was evaluated by immunoblot. PBS-washed cells were harvested three days post-miRNA transfection using 200 microliters 1X lysis buffer (Cell Signaling, Danvers, MA) with protease inhibitor cocktail (Roche, Indianapolis, IN) per well, and immunoblots were carried out. Primary antibodies were rabbit anti-Caspase 3 and mouse anti-Caspase 9 (#9665 and #9504, Cell Signaling, Danvers, MA)), mouse anti-alpha-tubulin (Sigma Aldrich, St. Louis, MO), anti-BCL2L11 (2933S, Cell Signaling, Danvers, MA), anti-β-actin (Santa Cruz Biotechnology, Santa Cruz, CA). Secondary antibodies were anti-mouse and anti-rabbit (Jackson ImmunoLabs, West Grove, PA,). Membranes were developed with Pierce ECL Western Blotting substrate (Rockford, IL) and exposed to film. Protein content of the samples was normalized to α-tubulin (51 kDa) or β-actin (43 kDa).

*Flow cytometry—* Total apoptotic cell percentage was evaluated using a dual marker flow cytometry kit. Cells were stained four to five days after miRNA transfection using the PE-Annexin-V Apoptosis staining kit I ( BD Pharmingen, San Diego, CA), as recommended.Three control cell samples (not treated with miRNAs) were plated one day prior to flow cytometry analysis and then treated with ten minutes of UV light one hour prior to staining. Cell forward and side scatter as well as Annexin-5 and 7-AAD positivity were collected with a FACScan and were analyzed using FlowJo software. Percent apoptotic cells are shown, reflecting a combination of singly- and doubly-staining cells per sample.
